# You Are Always on My Mind: Neural Synchrony Between Mothers and Their 2‐Year‐Olds During Collaborative Play

**DOI:** 10.1111/desc.70245

**Published:** 2026-07-06

**Authors:** Rebecca Terry, Victoria St Clair, Paola Pinti, Denis Mareschal

**Affiliations:** ^1^ Centre for Brain and Cognitive Development, School of Psychological Science Birkbeck University of London London UK

**Keywords:** collaboration, fNIRS, hyperscanning, neural synchrony, parent‐child interaction

## Abstract

Working with others is an essential skill for everyday life. Interpersonal neural synchrony (INS) has been suggested as a potential neurobiological correlate of collaboration between parents and school‐aged children. This study investigated whether interpersonal neural synchrony (measured using Wavelet Transform Coherence) is also a correlate of collaboration in young children, whose collaborative skills are still emerging. Data were collected from *N* = 20 mother‐child dyads with children aged 24–36 months old (mean age 30.15 months) using an fNIRS hyperscanning paradigm. Mothers and their children built Lego Duplo structures collaboratively and individually (separated by a curtain). Dyads showed neural coherence in different regions of the brain when building together and separately, possibly reflecting continued representations of their partner's actions, even when they could not see them. Greater neural coherence was found between the children's left prefrontal cortex (PFC) and the mothers’ right temporoparietal junction (TPJ) while working collaboratively. However, during the Individual condition, coherence was significantly greater between the children's right PFC and the mothers’ left TPJ. Furthermore, dyads were more likely to show overall greater neural coherence during the Individual than in the Collaboration condition if a second researcher was present in the testing session. This study demonstrates how INS can help us understand how mothers and children jointly engage in collaborative tasks. It also suggests a potential influence of others on mother‐child INS, which has not yet been explored.

## Summary

- Coherence was found between children's prefrontal and mothers’ temporoparietal regions, both when working together and individually.
- Mother‐child dyads were more likely to show greater coherence during the individual task if a second researcher was present.
- When only one researcher was present, dyads were more likely to show greater coherence during collaborative play.

## 1 Introduction

Human lives revolve around our interactions with others. This is particularly true in childhood, during which interactions with peers and caregivers scaffold a child's learning and development (Rogoff 1990). Collaborative interactions—in which the child works with a partner to achieve a common goal—are especially formative for very young children. Children start developing collaborative skills in the second and third year of life, as their social networks expand rapidly and their planning and mentalizing skills undergo substantial change (Frith and Frith 2003; Jung et al. 2015). In fact, 18‐ to 24‐month‐olds can complete cooperative tasks with an adult by learning parallel, then complementary, actions (Brownell et al. 2006; Warneken et al. 2006). In these interactions, the adult scaffolds the task for the child to help them complete it (Brownell 2011). However, it is not until around 3‐years‐old that children develop a more complex understanding of their own role in the collaborative interaction and show a desire to engage in extended collaboration with others (Gräfenhain et al. 2009; Hamann et al. 2012).

Children's desire to collaborate with others may emerge from earlier prosocial behaviours, such as helping, in which children start to recognise the goals and desires of others (Warneken and Tomasello 2007). Interestingly, both prosocial behaviour and successful cooperation in children have been linked to behavioural synchrony. Children who move synchronously and show more mutual smiling and eye contact during play are better at cooperating and show more prosocial behaviours (Rabinowitch et al. 2024; Tunçgenç and Cohen 2018). An association between behavioural synchrony and prosocial behaviour has been shown in children as young as 14 months old (Cirelli et al. 2014).

Behavioural synchrony is also important in mother‐child dyadic interactions (Feldman 2007). Behavioural synchrony refers to the temporal coordination of behaviours during an interaction, including eye‐gaze, positive affect, and vocalisations. Greater mother‐child behavioural synchrony has been associated with better cognitive processing in childhood, higher capacity for empathy and secure attachment between the mother and child (see Leclère et al. 2014, for a review). However, this research did not consider whether synchronous behaviours, which support cooperation and mother‐child interactions, reflect similarly synchronous patterns of brain activity in interacting participants.

It is therefore natural to ask whether brain‐to‐brain synchrony also plays a role during mother‐child collaboration. Hyperscanning techniques allow researchers to investigate the neural correlates of real‐time interactions between participants (Hakim et al. 2023) and allow for the quantification of the temporal alignment of brain activation between two or more people, known as interpersonal neural synchrony (INS). Functional near‐infrared spectroscopy (fNIRS) is one of the most common neuroimaging modalities in hyperscanning research (Hakim et al. 2023). fNIRS is based on neurovascular coupling principles of the brain, using light to measure changes in the concentration of oxyhaemoglobin (HbO) and deoxyhaemoglobin (HbR) that follow neuronal activity. This method is relatively low‐cost compared to MRI and can be used in a range of environments (Pinti et al. 2018, Pinti et al. 2020). fNIRS is particularly suited to studying developmental populations, as it has a higher tolerance for motion artifacts than MRI or EEG (Lloyd‐Fox et al. 2010). Additionally, the development of wireless fNIRS systems has allowed for measurement during naturalistic tasks. Using fNIRS for hyperscanning takes advantage of this low cost and ease of use to record data from multiple interacting participants.

It is theorised that INS may reflect individuals simultaneously controlling their own behaviour and predicting others' behaviours during interactions, resulting in similar patterns of brain activation across time (Hamilton 2021). The *relational neuroscience* framework suggests that greater INS can be correlated to two factors: *interpersonal closeness* and *interactivity* (De Felice et al. 2025). For example, romantic couples and family members commonly show greater INS compared to strangers completing the same task (for a review, see Zhao et al. 2024).

Mother‐child dyads have also been found to show greater INS than adult stranger‐child dyads during cooperative tasks (Reindl et al. 2022). INS has been found between mothers and their children using fNIRS hyperscanning in a range of interactive tasks, including co‐watching of videos and free play (Liu et al. 2024; Nguyen, Abney, et al. 2021; Papoutselou et al. 2024), which may help to facilitate social bonding (Hoehl et al. 2021; Nguyen, Abney et al. 2021). Greater parent‐child INS during cooperative tasks has also been related to greater child emotion regulation (Reindl et al. 2018). Taken together, this body of research suggests a key role of mother‐child INS in infancy and later childhood development. However, there is a lack of knowledge about how INS relates to mother‐child interaction and child development during the toddler years.

fNIRS hyperscanning studies have found greater INS in the bilateral prefrontal cortex (PFC) and temporoparietal junction (TPJ) in collaborative tasks with mother‐child pairs (St Clair et al. 2025; Liu et al. 2016; Nguyen et al. 2020). In the study by Nguyen et al. (2020), dyads of 5 to 6‐year‐old children with their mothers completed tangram puzzles together and individually, and showed greater INS when working collaboratively. Greater INS was also associated with collaborative success. The dorsolateral PFC is associated with task switching, planning, and preparing for actions (Hyafil et al. 2009; Pochon et al. 2001)—processes which are required for participants to work cooperatively. Greater INS in the TPJ is also often found during cooperation (Czeszumski et al. 2022). The TPJ has been implicated as part of the mentalising network of the brain in single participant studies (Frith and Frith 2006; Pochon et al. 2001; Saxe and Kanwisher 2003), in addition to being associated with theory of mind tasks and inferring others’ goals (Van Overwalle 2009). Similar patterns of greater INS in the PFC and TPJ during cooperation were found in father‐child dyads (Nguyen, Schleihauf, et al. 2021) and dyads with 4‐ to 6‐year‐old children (St Clair et al. 2025), indicating that neural synchrony could be a neurobiological correlate of successful collaboration in children over 4 years of age.

By age 4, most children in the UK have started school and are more familiar with working collaboratively with others. In children under 3‐years‐old, the socio‐cognitive skills underpinning successful collaboration are still rapidly developing and their success and desire to collaborate vary greatly. Because of this, it is unclear whether INS emerges during collaboration between parents and children under 3‐years‐old. In one of the only fNIRS hyperscanning studies with mother‐child pairs aged 1‐ to 3‐years‐old, Morgan et al. (2023) found that greater INS was associated with matching on high positive affect during a 3‐minute play task. This study demonstrates that fNIRS hyperscanning can be conducted with toddlers; however, the short task without a control condition makes it difficult to compare the findings to those with older children.

In the present study, we aimed to bridge the gap between fNIRS hyperscanning studies of mother‐infant dyads and collaboration research with school‐age children, to investigate whether INS is a correlate of collaboration in mother‐toddler dyads. As in the paradigm used by Nguyen et al. (2020) and St. Clair et al. (2025), mother‐child dyads completed a task together and individually, while brain activity was simultaneously measured using fNIRS in the bilateral prefrontal and temporoparietal areas. These areas were chosen because of their association with planning and the mentalising network (Frith and Frith 2006; Saxe and Kanwisher 2003) and INS in mother‐child pairs during collaboration with older children (Nguyen et al. 2020). The primary aim of the current study was to compare mother‐toddler INS between conditions in which the dyad either worked collaboratively or worked independently. We hypothesised, based on previous research by Nguyen et al. (2020) and St Clair et al. (2025), that mother‐toddler dyads would exhibit greater levels of INS when working together compared to when working individually.

## 2 Method

### 2.1 Participants

Twenty‐nine mother‐child dyads took part in this study. Nine dyads were excluded from the analysis: *N* = 7 because of insufficient data (including the child refusing or removing the fNIRS cap) and *N* = 2 because of technical difficulties.

The final *N* = 20 dyads included in this study consisted of children aged between 25 and 35 months (mean age 30.15 months, 11 female) and their biological mothers aged between 32 and 50 years (mean age 38.05 years). The sample was drawn from a mixed urban and suburban population from London, UK. Six dyads did not disclose demographic information, and 3 mothers did not disclose their education level. For the children included, 71% identified as White, 14% Asian and 14% from a Mixed background. For the mothers, 59% held a post‐graduate qualification, 29% held a first degree or equivalent, and 12% held an A‐level or equivalent qualification.

All children were typically developing with no known neurological conditions, and all participants had normal or corrected‐to‐normal vision. Families were recruited from the pre‐existing Birkbeck Centre for Brain and Cognitive Development (CBCD) database. Travel expenses were reimbursed, and all children received a t‐shirt and a certificate to thank them for taking part. Mothers provided written informed consent for themselves and their children. The procedures for this study were approved by the Birkbeck School of Psychological Science Ethics Committee (certificate number 2324042).

### 2.2 Materials

Prior to testing, mothers completed two questionnaires: the Mother Object Relation Scale (MORS‐Child, Simkiss et al. 2013) and Ages and Stages 3^rd^ Edition (ASQ‐3, Squires et al. 2009)—details of which are available in the supplemental material.

### 2.3 Procedure

Testing took place in the Preschool Lab at the *Birkbeck ToddlerLab*, a semi‐naturalistic laboratory designed to look like an early years classroom. Families attended a single session. After arriving, children played while the study was explained to their mothers. Children also completed an inhibition and a helping task (described further in the supplementary materials) before being fitted with fNIRS caps.

For 12 of the 20 sessions, a second researcher assisted with warm‐up play with the child and with the collaborative building task. For the other 8 sessions, all data collection was carried out by the primary researcher only.

#### 2.3.1 Collaborative Building Task

**FIGURE 1:**
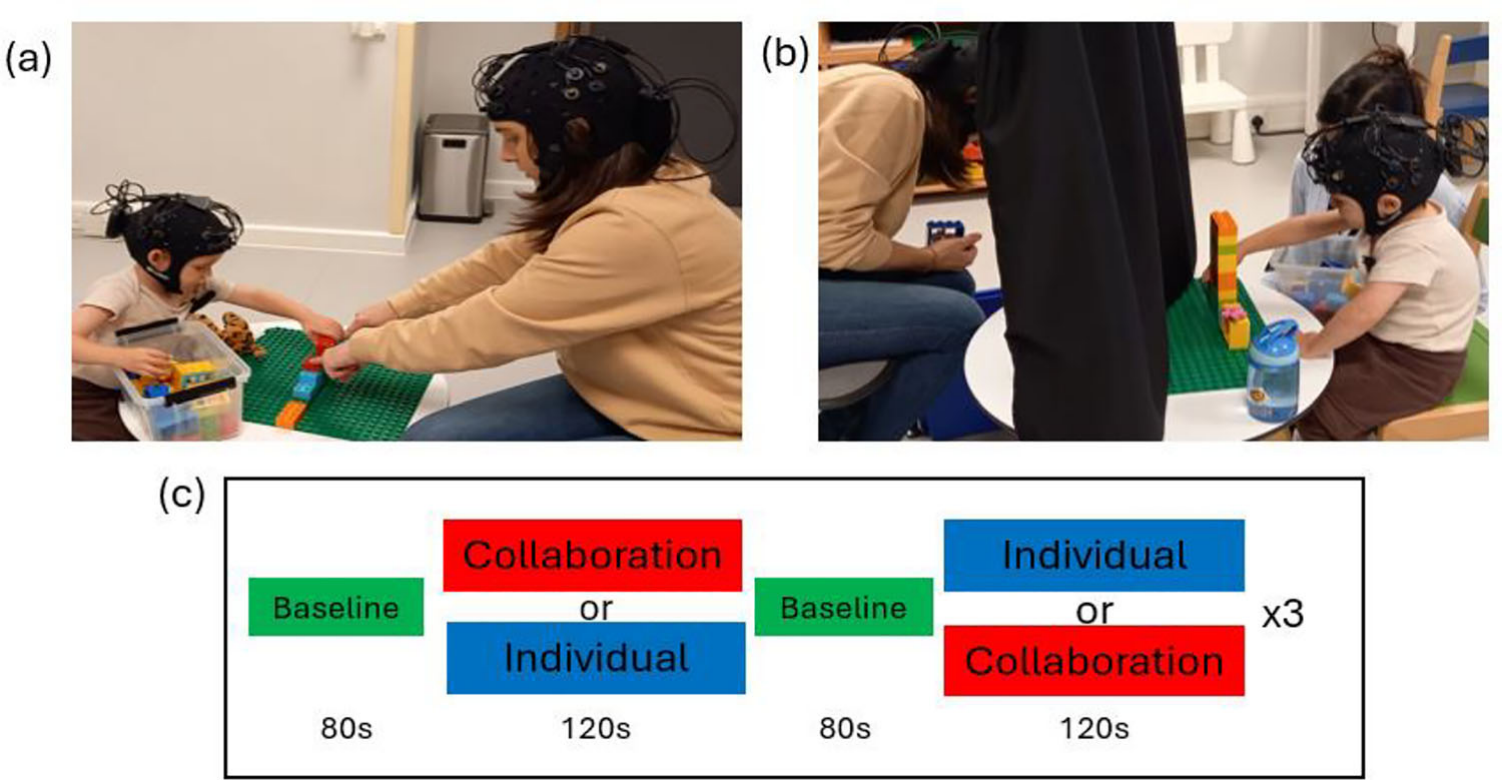
Collaborative building task. (a) Collaborative building condition. (b) Individual building condition. (c) Structure of the building task—dyads were counterbalanced on the start condition, and conditions were repeated 2 or 3 times.

In the collaborative building task, the mother and child sat at a small child‐sized table facing each other with a curtain in between, which could be pulled back and forth to separate the pair (see Figures 1a and 1b). The building task consisted of 3 trials of each condition (Collaboration and Individual), each lasting 120s separated by an 80s baseline block (Figure 1c). Dyads were included if they completed at least 2 trials of each condition. The dyads alternated between the Collaboration and Individual conditions. Condition order was counterbalanced across dyads.

Participants were asked to build a single structure out of Lego Duplo. In the Collaboration condition, participants were instructed to build one structure together, interacting as they would at home (see Figure 1a). In the Individual condition, participants were separated by a fabric curtain and asked not to talk to each other while each built their own structure (see Figure 1b). For the baseline phase, children sat with their mother and watched drone footage of natural scenes (e.g., waterfalls, mountains) on a large screen.

### 2.4 Data Acquisition

fNIRS, video and audio data were collected during the collaborative building task (in both the Collaborative and Individual conditions). Video recordings were also collected during the inhibition and helping tasks. Behavioural videos were recorded using iVMS 4200 cameras (Hikvision, China) with 4 viewpoints, and audio was recorded using clip‐on microphones on the mother and child.

#### 2.4.1 fNIRS Data Acquisition

Two wireless fNIRS systems (Brite MKII, Artinis Medical Systems, The Netherlands) were used to measure HbO and HbR concentration changes for each dyad. The regions of interest (ROI) identified for this study were the left and right dorsolateral prefrontal cortex (PFC) and left and right temporoparietal junction (TPJ), related to action planning and preparation and the mentalising network (Frith and Frith 2006; Saxe and Kanwisher 2003). These regions and the positioning of the channels were based on previous child hyperscanning studies (St Clair et al. 2025; Nguyen et al. 2020). Participants’ heads were measured to ensure cap fit, and the 10–20 system was used to ensure reliable cap placement between participants. The FPz point was aligned to just above the bridge of the nose, with the edge of the cap parallel to the eyebrows.

**FIGURE 2:**
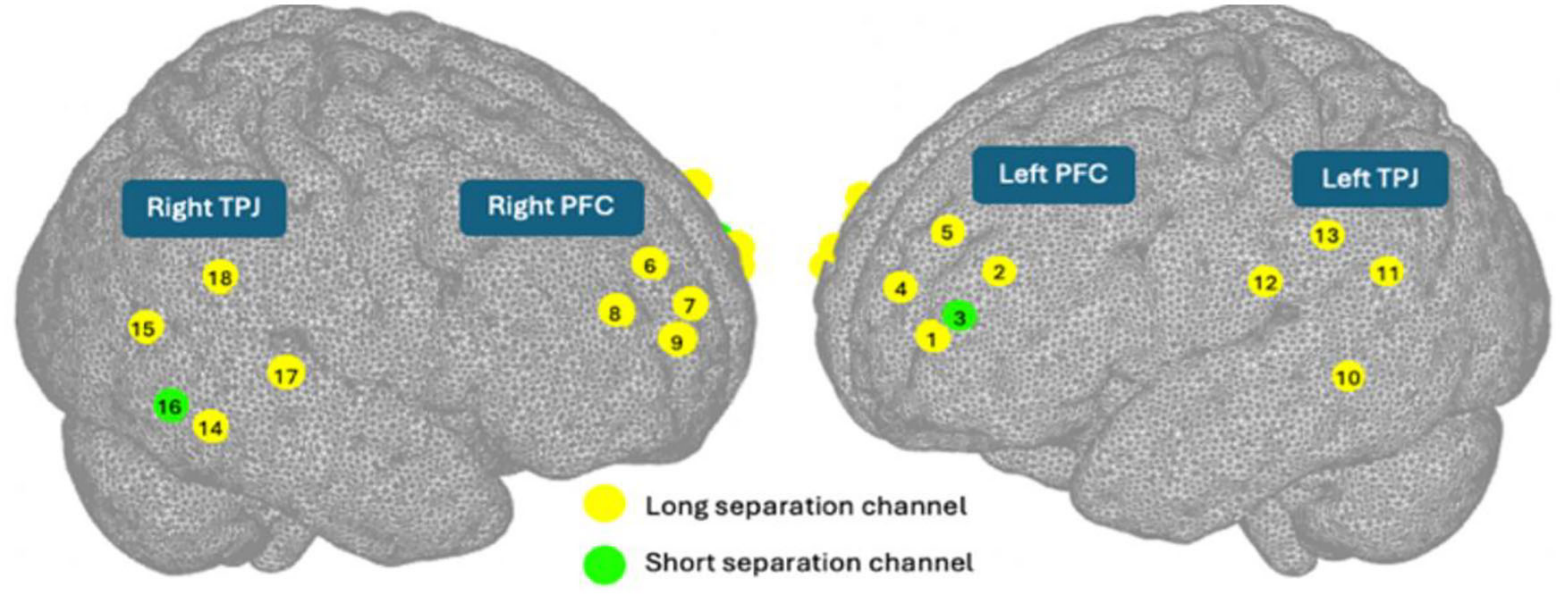
Optode configuration for both mother and child. Figure adapted from St Clair et al. (2025), Imaging Neuroscience, CC BY 4.0. Yellow = long separation channel, green = short separation channel.

Each participant's cap was equipped with 16 long‐separation and 2 short‐separation channels (SSCs) derived from 10 light sources and 8 detectors over the left and right PFC and TPJ areas (wavelengths 760 and 850 nm, sampling frequency of 25 Hz, Figure 2). A source‐detector separation of 3 cm was used for the long‐separation channels for the mother and 2.5 cm for the child. For each participant, the SSCs (source‐detector distance of 1 cm) were included in the right TPJ and left PFC regions.

### 2.5 Data Analysis

fNIRS preprocessing and wavelet transform coherence (WTC) analysis were conducted in MATLAB (MathWorks) using the analytical pipeline developed by St. Clair et al. (2025) and following guidelines for parent‐child INS measurement (Nguyen, Hoehl, and Vrtička 2021). Multi‐level modelling was also conducted in MATLAB, with additional statistical analysis in IBM SPSS (V29).

#### 2.5.1 fNIRS Pre‐Processing

A visual inspection was performed on the raw voltage data in both the time and frequency domains. Any channels that did not show a clear heart rate peak, were saturated, or had poor optical coupling were excluded from the analysis. The remaining data was processed using MATLAB functions from Homer2 (Huppert et al. 2009). First, raw intensity data were converted into optical density (*hmrIntensity2OD* function). Motion artifacts were corrected using the wavelet motion correction method (Molavi and Dumont 2012), with an interquartile range of 0.8 for the child and 1.5 for the mother (*hmrMotionCorrectWavelet*). Optical density data were filtered with a band‐pass filter in the range [0.01 0.5] Hz (*hmrBandpassFilt*) and converted into HbO and HbR concentration values using the Beer‐Lambert law with DPF values of [6 6] for the mother and [5.4 4.6] for the child (*hmrOD2Conc*). Signal from SSCs was regressed out of long‐separation channels to minimise the effect of physiological interference on the estimation of the haemodynamic activity of interest for analysis (St Clair et al. 2025; Pinti et al. 2024). SSC data was available for *N* = 10 mothers and *N* = 5 children, all of whom only had 1 valid SSC. When present, the SSC signal was regressed out of all long‐separation channel signals before WTC analysis. When no SSC data was available, WTC analysis was performed on the unregressed signal. SSC regression was performed for all participants with a valid SSC.

#### 2.5.2 WTC Analysis

WTC was used to measure INS between the mother and child. This method provides a measure of the correlation between the fNIRS time series data of the dyads for each channel as a function of frequency and time (for more information, see Hakim et al. 2023). Coherence was calculated for each combination of valid channels from the mother and child for HbO and HbR, and averaged across the frequency band of interest (0.02–0.1 Hz) identified for collaborative hyperscanning tasks with mother‐child dyads (St Clair et al. 2025; Nguyen et al. 2020). An overarching WTC analysis was conducted across the entire task before WTC values were cut out and averaged within each 120s condition block. Coherence values from each trial were averaged to calculate channel‐wise mean coherence values for the Collaboration and Individual conditions.

### 2.6 Statistical Analyses

#### 2.6.1 Pseudo‐Dyad Analysis: Comparison of Coherence for True vs Pseudo‐Dyads

A pseudo‐dyad control analysis was run using the method developed by St Clair et al. (2025). For this analysis, a fast Fourier transform was performed on the mother's data to extract the magnitude and phase of the time series at each sampling point. The phase data were randomised (effectively jittering the timeseries locally) before an inverse Fourier transform was used to convert the data back to the original form, leaving the frequency information unchanged but locally disrupting temporal information. A WTC analysis was then performed between the phase‐randomised pseudo‐mother data and the original child data, using the method described above. Phase‐scrambling and subsequent WTC analysis were run with 100 iterations for each dyad. An average was then taken of the coherence values to give a mean pseudo‐dyad coherence for each channel combination for all dyads. Linear mixed‐effect models were constructed for both HbO and HbR with WTC as the outcome, with fixed effects of Dyad Pairing (true vs pseudo‐dyads), Condition, ROI and Dyad Pairing × Condition × ROI, with a random effect of dyad. Models were fit using the maximum likelihood method.

#### 2.6.2 Aim 1: Comparison of Coherence Between Conditions for HbO and HbR

Linear mixed‐effect models were constructed using the channel‐wise coherence values in separate models for HbO and HbR. The models included all available WTC values from homologous and non‐homologous channels from the mothers and children. Each model contained a random effect of dyad, and the outcome was always WTC. Condition, ROI, and an interaction of Condition × ROI were entered as fixed effects. Channel‐wise data was nested into 16 ROI groups, representing all possible combinations of the mothers’ and children's ROIs, shown in Table S1. Models were fit using the maximum likelihood method. Experimental models were compared to the null model of Coherence ∼ 1 + (1|Dyad), using ANOVAs with a Satterthwaite approximation for degrees of freedom.

To identify differences in coherence between conditions for specific combinations of mothers’ and children's brain regions, planned paired *t*‐tests were performed. Sixteen separate paired *t*‐tests were performed for both HbO and HbR to compare all possible combinations of the mothers’ and children's ROIs (4 for each participant). For each region of interest grouping, we performed a paired *t*‐test between the channel‐wise WTC values for the Collaboration and Individual conditions of all channels that fell within that region. The *t*‐tests were FDR‐corrected for multiple comparisons (Benjamini and Hochberg, 1995).

#### 2.6.3 Aim 2: Exploratory Analysis of Inter‐Dyad Variability in HbO Coherence

During the primary analyses, for the HbO data, we noticed a large amount of inter‐dyad variability in which condition they showed greater coherence. We therefore conducted an additional set of analyses to further explore this variability.

A ‘Condition Score’ was calculated for each dyad, which indicated the proportion of channels that showed greater coherence during the Collaboration condition. Condition Scores ranged from 0 to 1, with 1 indicating that all channels for the dyad showed greater coherence during the Collaboration condition and 0 indicating that all channels showed greater coherence during the Individual condition. A multiple linear regression was performed with Condition Score as the outcome. Child age, gender and MORS questionnaire results were entered as predictors (see supplement for further detail on MORS questionnaires). As some sessions involved multiple researchers, the presence of a second tester was also entered as a predictor.

## 3 RESULTS

### 3.1 Comparison of Coherence for True vs Pseudo‐Dyads

The following experimental model was used to investigate differences in coherence between true dyads and phase‐scrambled pseudo‐dyads:

i. M1: Coherence ∼ Dyad Pairing + Condition + ROI + Condition x DP x ROI

For HbO, there were significant fixed effects of the Dyad Pairing x ROI (F(15,5840) = 2.62, *p* < 0.001) and Condition x DP x ROI (F(15,5840) = 2.65, *p* < 0.001). These findings indicate that for some regions of the brain, there was a difference in coherence between true and pseudo‐dyads, between conditions.

For HbR, there was a significant fixed effect of Dyad Pairing only (F(1,5840) = 7.24, *p* = 0.007). These results suggest that, unlike HbO, HbR coherence for true dyads was generally greater than for pseudo‐dyads across all regions and conditions.

### 3.2 Comparison of Coherence Between Conditions for HbO and HbR

#### 3.2.1 HbO

The following experimental models were compared to the null model (M0): Coherence ∼ 1 + (1|Dyad) in a stepwise method. Full estimations of fixed effects can be found in the supplementary tables, with channel inclusion results.

i. M2: Coherence ∼ Condition + (1|Dyad)

Adding a fixed effect of condition did not significantly improve the model compared to the null model (*χ^2^* (1) = 2×10^−5^, *p* = 0.999). There was no significant difference in coherence across the whole brain between conditions—*t*(1459) = ‐0.001, *p* = 0.99.

ii. M3: Coherence ∼ Condition + ROI + Condition x ROI + (1|Dyad)

Model 3 was a significant improvement over the null model (*χ^2^* (31) = 71.7, *p* < 0.001). Within M3, the effect of condition was not significant (*F*(1, 2920) = 0.37, *p* = 0.54). Effects for ROI (*F*(15, 2920) = 3.10, *p* < 0.001) and Condition x ROI were significant (*F*(15, 2920) = 3.37, *p* < 0.001). The same results were found when the models were run using a Fisher z transformation to account for the beta‐like distribution of the WTC values.

Separate paired *t*‐tests were run using the channel‐wise data for each ROI combination for the mothers and children (4 ROIs each, giving a total of 16 combinations) to identify regions with significant differences between conditions.

**FIGURE 3:**
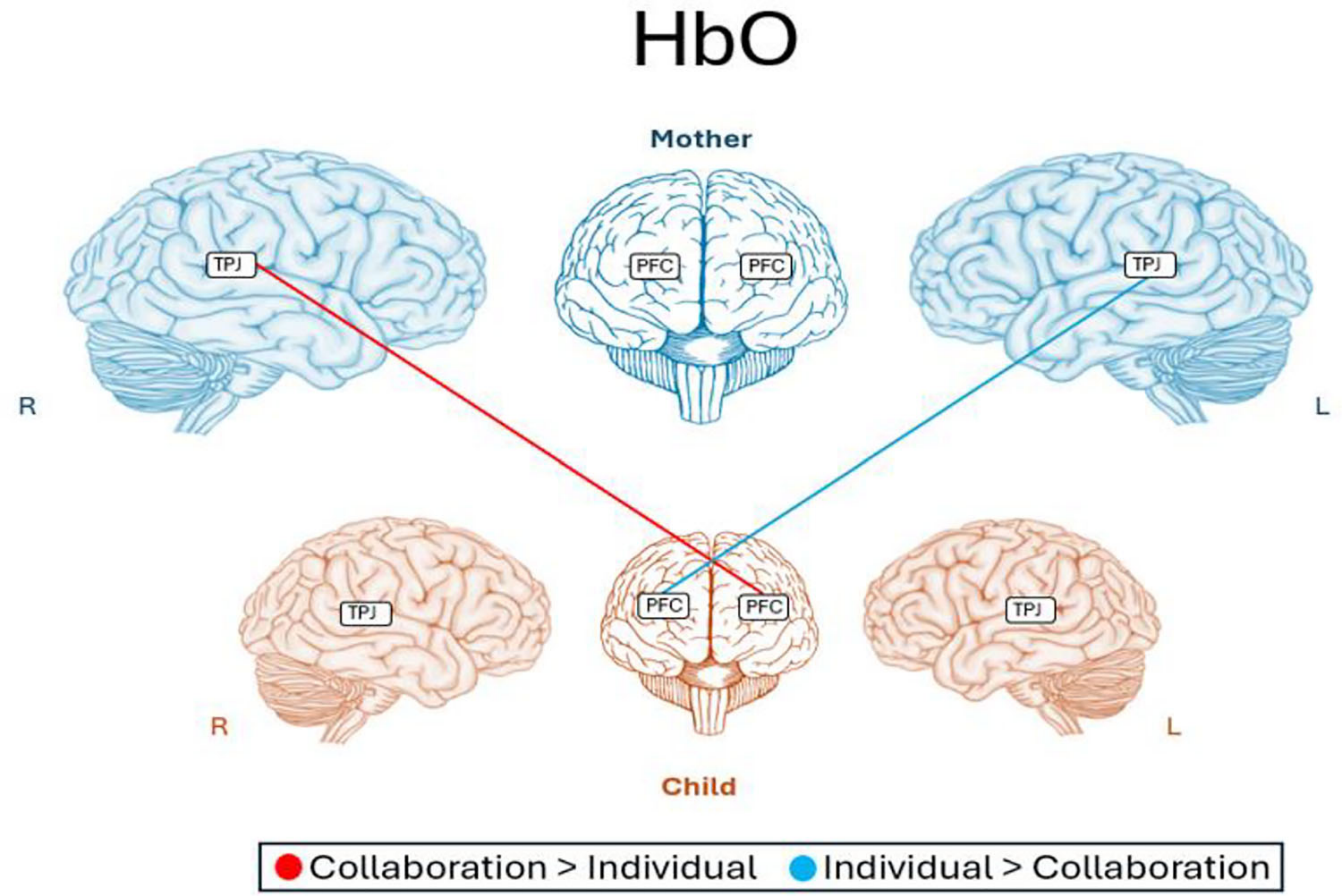
Regions of the mothers’ (blue) and children's (orange) brains that showed significant differences in coherence between conditions for oxyhaemoglobin (HbO). Red lines indicate regions that showed greater coherence in the Collaboration condition, and blue lines indicate greater coherence in the Individual condition (FDR corrected *p *< 0.05). PFC, prefrontal cortex; TPJ, temporoparietal junction.

The paired *t*‐tests showed coherence differed significantly by condition in three region combinations, though only two remained significant after FDR correction. Coherence between children's left PFC and mothers’ right TPJ was greater during Collaboration than Individual (*t*(67) = 3.51, *p_adj_* = 0.006). Coherence between children's right PFC and mothers’ left TPJ was stronger during the Individual than Collaboration condition (*t*(89) = −4.44, *p_adj_* < 0.001), shown in Figure 3.

#### 3.2.2 HbR

Mixed effect modelling was conducted using the same method on the HbR data, with experimental models compared to the null model (M0) of: Coherence ∼ 1 +(1|Dyad)

iii. M2: Coherence ∼ Condition + (1|Dyad)

The addition of a fixed effect of condition significantly improved the model over the null model *χ^2^* (1) = 5.37, *p* = 0.021 (full estimates in supplement). In the HbR data, there was significantly greater coherence across the whole brain in the Individual condition compared to Collaboration—*t*(1459) = −2.24, *p* = 0.025.

As with HbO, paired *t*‐tests were run to identify combinations of brain regions for the mothers and children that showed a significant difference in coherence between conditions for HbR. There was significantly greater coherence during the Individual condition between the children's right PFC and the mothers’ right PFC and right TPJ. However, these findings did not survive correction for multiple comparisons.

### 3.3 Exploratory Analysis of Inter‐Dyad Variability in HbO Coherence

A Condition Score was calculated for each dyad to indicate the proportion of channels for that dyad that showed greater coherence during the Collaboration condition, with 1 indicating that all channels showed greater coherence during Collaboration and 0 indicating that all channels showed greater coherence during the Individual condition.

**FIGURE 4:**
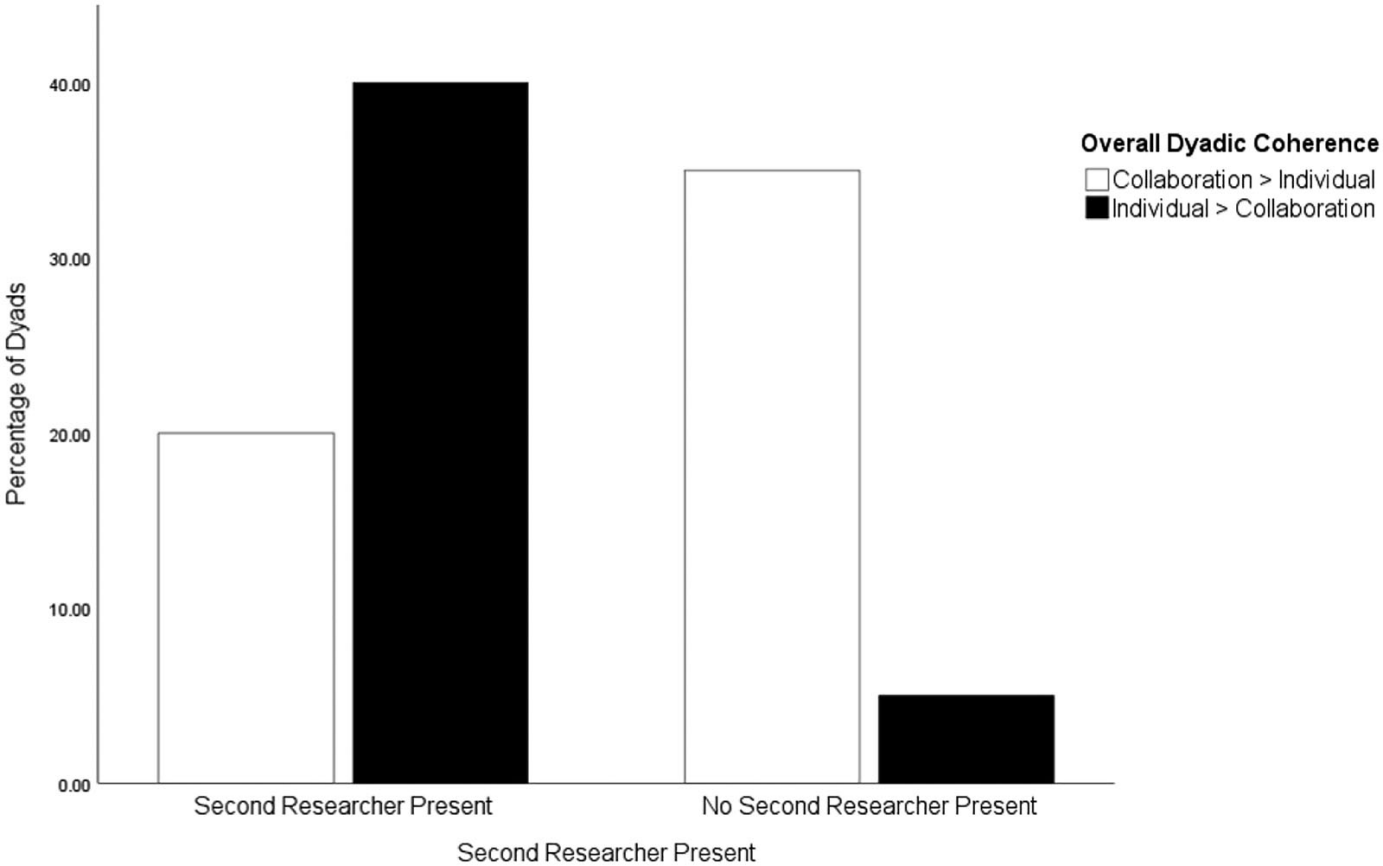
Bar chart showing the significant association between the condition dyads show greater coherence in and the presence of a second researcher.

An exploratory multiple linear regression found that Condition Score was not significantly predicted by child age, gender or MORS questionnaire scores. Condition Score was significantly predicted by the presence of a second tester (F(1,19) = 8.03, *p* = 0.011). A Fisher's exact test revealed that dyads were more likely to show greater coherence in the Individual condition over Collaboration when there was a second researcher present in the session, as shown in Figure 4.

## 4 DISCUSSION

This study investigated INS (as measured by WTC) between 2‐year‐olds and their mothers in a collaborative compared to an individual task. When measured across the whole brain, there were no condition‐related differences in HbO. However, condition‐related differences in coherence were revealed in region‐of‐interest comparisons. As hypothesised, neural coherence was stronger during the Collaboration condition than the Individual condition, but only between the children's left PFC and the mothers’ right TPJ. In contrast, coherence was stronger during the Individual than the Collaborative condition between the children's right PFC and mothers’ left TPJ.

Interestingly, neural coherence also appeared to be influenced by the presence of other adults in the testing session. Mother‐child dyads were more likely to show greater coherence during the Individual condition in sessions with a second researcher present. However, when there was no second researcher present, dyads were more likely to show the hypothesised result of greater coherence during the Collaboration condition.

### 4.1 Comparison of Coherence Between Conditions for HbO and HbR

HbO and HbR analyses revealed differences in coherence between conditions. When analysing HbR, we found that across the whole brain, neural coherence was significantly higher in the Individual condition compared to the Collaboration condition. This finding was unexpected, as it stands in contrast to previous studies that showed greater coherence in HbR during interactive over individual task contexts (St Clair et al. 2025; Hirsch et al. 2017, Hirsch et al. 2018). These findings demonstrate the importance of reporting coherence in both chromophores to improve the interpretation of fNIRS results. The unexpected level of coherence found in the current study may reflect a continued interaction between the dyad or concern over the other's actions when separated (see further discussion below). However, it may also be that no significant difference in HbO coherence between conditions was found across the whole brain because of the relatively small sample size. While the sample size of the current study was comparable to previous developmental hyperscanning studies (Leong et al. 2017; Piazza et al. 2020), future studies with larger samples can establish whether the patterns reported here are robust and replicable.

#### 4.1.1 Cross‐Region Coherence During Dyadic Interaction

The coherence shown between the mothers’ TPJ regions and children's PFC regions suggests that although they were completing the same task, they may be engaging in the task in different ways. In both conditions, the building task was unconstrained and designed to engage the toddlers for the length of the task. However, it meant that the dyads were not assigned a clear, pre‐set goal. This is in contrast to other studies with mother‐child dyads, who had to match tangram puzzles to templates (St Clair et al. 2025; Nguyen et al. 2020). The Lego task used here did not require much cognitive resource for the mother, and they likely did not feel pressure to complete a specific objective. Because of this, mothers may have considered their Lego building less important than monitoring their child's mental state. Interpretation of others’ mental states from their actions has been associated with activity in the TPJ (Arioli et al. 2023; Van Overwalle 2009). In contrast, the children were engaged in the building task, requiring more planning and working memory than for the mothers, which is supported by the PFC (Moriguchi and Hiraki 2013; Nitschke et al. 2017). During the task, both the mother and child would have been observing and predicting the other's behaviour and planning their own actions, but potentially for different purposes. It could be that mothers were focused on interpreting their child's state and ensuring their continued well‐being. In contrast, children may have been more focused on successfully building the Lego structure, which could explain coherence between the TPJ in the mother and the PFC regions in the child. It is important to note that our analyses focused on the regions that exhibited interactions between the mother and child, rather than those that showed the highest activation for the mother and child separately, although one might speculate that the cross‐brain coherence could reflect that the joint interaction was driven by different objectives for the mother and child.

There is some debate as to whether coherence in analogous regions of the brain (e.g., child left TPJ to mother left TPJ) is simply caused by shared sensory input (Novembre and Iannetti 2021). In this study, cross‐region coherence was stronger than in analogous regions. It may be that cross‐region coherence reflects higher‐order processing of actions, such as creating shared goals and intentions, compared to simply completing the same task at the same time. This conclusion is supported by the results of the pseudo‐dyad analysis. Pseudo‐dyads with disrupted temporal information and retained frequency information showed significantly lower coherence than true dyads. This suggests that coherence is related to direct interpersonal interaction and not simply task‐related activity, as also reported by St. Clair et al. (2025).

#### 4.1.2 Hemispheric Differences in Coherence Between Conditions

As with older children (St Clair et al. 2025; Nguyen et al. 2020), this study found greater neural coherence between mothers and their children when they worked together than when completing the same task individually. However, following corrections for multiple comparisons, coherence was only significantly greater than chance between the children's left PFC and the mothers’ right TPJ. Unexpectedly, coherence was stronger in the Individual condition than in the Collaboration condition between the children's right PFC and the mothers’ left TPJ. In both conditions, coherence may reflect the mother and child continuing to represent each other mentally, even when separated. Although a curtain was drawn between the mother and child during the Individual condition, they could still hear each other and the children often tried to engage with their mothers by talking through the curtain or reaching their hands underneath. INS has been shown to occur during interactions with no visual contact (St Clair et al. 2025; Osaka et al. 2015), so these small interactions may have led to the unexpected coherence found in the Individual condition. It may also be that mothers were thinking about the actions of their child, regardless of the experimental condition. Similar results were shown by Papoutselou et al. (2024), who found no difference in mother‐led synchrony between interactive and individual tasks.

The hemispheric differences in the regions showing greater coherence between the Collaboration and Individual conditions may reflect the continued mother‐child interaction relying on different perceptual information available to the dyad. A recent meta‐analysis found that action observation tasks were associated with activation in the right TPJ, whereas the left TPJ was recruited for tasks involving the interpretation of beliefs and mental states (Arioli et al. 2023). It could be that, during the Collaboration condition, mothers could see their children and use their actions to interpret their behaviour, possibly recruiting the right TPJ. In the Individual condition, the mother could not see her child and would have had to rely on more abstract cues, such as noises, second researcher conversations with the child and previous knowledge of the task to interpret her child's actions. This might recruit the left TPJ. In the prefrontal cortex, the left PFC has been associated with language processing and the right with visuospatial processing (Floel et al. 2004). The finding of coherence in the children's left PFC during the Collaboration condition and right PFC coherence in the Individual condition may reflect the child processing the mother's guidance when working together, but requiring more visuospatial processing when building alone.

### 4.2 Exploratory Analysis of Inter‐Dyad Variability in HbO Coherence

There was a high level of inter‐dyad variability in HbO coherence observed. Some dyads showed stronger coherence in the Collaboration condition and others in the Individual condition. None of the additional variables measured predicted this variance. Instead, we found a significant association between the presence of a second researcher in the session and dyads showing greater coherence in the Individual condition.

During testing, the primary researcher was required to ensure the smooth running of the study and the quality of the NIRS data collection, and so they interacted less with the dyad. In contrast, when a second researcher was present, they interacted more with the child to ensure their continued well‐being and compliance. The second researcher, when present, would usually stay close and encourage the child during the Individual condition (seen in Figure 1b), which was often necessary to complete the procedure. Mothers would have heard the other adult interacting with their child. It could be that this increased her concern over her child's actions, leading to increased INS. However, when only one researcher was present, they would have interacted with the child less, and so did not cause as much maternal concern. This may support findings from Nguyen et al. (2024) that more synchrony may not always be better.

It could also be that when the second researcher interacted with the child in the Individual condition, they talked more than parents did in the Collaboration condition, driving an increase in the shared perceptual processing of auditory information, and therefore of coherence, in Individual compared to Collaboration. One might also speculate that when approached by a researcher who was a stranger to the child, the child may have sought a secure base (similar to Ainsworth and Bell's (1970) Strange Situation). As the children were discouraged from interacting with their mother during the Individual condition, they may have tried to imagine what she was doing instead.

When a second researcher was present in the session, their interaction with the child may have distracted both the mother and child, leading to unexpected mutual prediction of behaviour, an increase in shared perceptual information, or both, and therefore increased neural coherence compared to when there was only one researcher present. Similarly, Lu et al. (2019) found increased INS between two people when a confederate praised participants compared to when they received no feedback, a finding that supports the idea that those in the environment of an interaction can influence INS of a dyad. In sessions with only one researcher present, dyads were more likely to show greater INS in the Collaboration condition, in line with previous research (Nguyen et al. 2020) and our original hypothesis. These findings may indicate methodological considerations that can lead to unexpected outcomes in hyperscanning studies of toddlers.

### 4.3 Limitations and Future Research

In this study, we discovered an association between the presence of a second researcher and dyads displaying greater overall coherence in the Individual condition. In developmental research, especially with young children, it is common to have multiple researchers in a testing session to ensure the child is happy and the study runs smoothly. However, this is not often considered when comparing findings from young children with older children or adults. Adults and children also act differently when they know they are being observed (Fujii et al. 2015; Hamilton and Lind 2016). A limitation of this study and hyperscanning research generally is that it focuses on how the interactions between the dyad alone relate to INS, without considering the interactions that are also happening with the researchers and the wider social environment of the study. A possible avenue for future research could be to directly test the influence of researchers and confederates on dyadic INS. This may also allow us to quantify the effect of background environmental measures, such as researcher speech, in eliciting INS as a shared perceptual source.

Another limitation of this study is that it focused primarily on neural synchrony between the mother and child and did not consider potential physiological synchrony. Parents and children show physiological synchrony across developmental stages in measures such as heart rate and cortisol level in stressful tasks (Davis et al. 2018; Hibel et al. 2015), which facilitates attachment (Feldman 2017). It would be useful for future research to measure behavioural, physiological and neural synchrony, to provide a broader perspective on how mother‐child interactions change from infancy to early childhood.

Furthermore, the building task used was designed to be unstructured to engage the child and allow for naturalistic interactions; however, it may not have been engaging enough for the mother. This might explain the unexpected results of coherence emerging in both the Individual and Collaboration conditions. Future studies should ensure tasks are engaging for both the mother and child, with a clear goal, to allow for better comparisons between conditions.

### 4.4 Conclusions

INS in mother‐toddler pairs is varied and likely influenced by the task and environment. As in studies with older children, neural coherence is found during mother‐toddler collaboration. Contrary to previous studies, coherence is also found when mothers and toddlers are visually separated and working individually. One might speculate that this reflects mothers’ concern about what their child is doing in a new environment, especially when they cannot see them. Mothers may mentally represent what the child is doing, relying on sound and previous knowledge, rather than focusing on completing their own task. This effect is even more pronounced when the child is interacting with a strange adult. This study highlights the importance of investigating INS in mother‐child interactions as a possible route to understanding how individuals may approach a joint task in different ways, since differences in engagement may not be observable at the behavioural level. This study also reveals some of the methodological challenges of collecting fNIRS hyperscanning data from young children.

## Author Contributions

**Rebecca Terry**: writing – original draft, writing – review and editing, formal analysis, visualization, investigation, conceptualization, methodology, data curation, supervision, project administration. **Victoria St Clair**: writing – review and editing, software, formal analysis, validation, visualization. **Denis Mareschal**: funding acquisition, supervision, project administration, writing – review and editing, conceptualization, methodology, resources. **Paola Pinti**: supervision, writing – review and editing, conceptualization, methodology, software, funding acquisition, formal analysis, project administration, resources.

## Funding

This work was funded by Leverhulme Trust grant RPG‐2021‐280. Additional support was provided by a Wellcome Trust multi‐user equipment grant (Grant No. 212979/Z/18/Z).

## Ethics Statement

All study procedures were approved by the Birkbeck School of Psychological Science Ethics Committee (certificate no. 2324042).

## Conflicts of Interest

The authors declare no conflicts of interest.

## Supporting information




**Supporting Information: Table S1**: Grouping of Channels for Region of Interest Analysis. **Table S2**: Long channel inclusion data for the mother and child. **Table S3**: Estimates of fixed effects for the HbO model M2: Coherence ∼ Condition + (1|Dyad). **Table S4**: Estimates of fixed effects for the HbO model M3: Coherence ∼ Condition + ROI + Condition x ROI + (1|Dyad). **Table S5**: Estimates of fixed effects for the HbR model M2: Coherence ∼ Condition + (1|Dyad). **Table S6**: Descriptive statistics for all additional participant variables. **Table S7**: Estimates of fixed effects for the HbO model M2.1: Coherence ∼ Condition + Child Age + (1|Dyad). **Table S8**: Estimates of fixed effects for the HbO model M2.2: Coherence ∼ Condition + MORS Invasiveness Score + (1|Dyad). **Table S9**: Estimates of fixed effects for the HbO model M2.3: Coherence ∼ Condition + Helping Score + (1|Dyad). **Table S10**: Estimates of fixed effects for the HbO model M4: Collaboration Coherence ∼ Inhibition Score + (1|Dyad). **Figure S1**: Factors that significantly predicted dyadic coherence across conditions and in Collaboration only. (a–c) Dyadic coherence across conditions was significantly predicted by (a) Child Age, (b) MORS Invasiveness Score and (c) Child Helping Score. (d) Dyadic coherence during the Collaboration condition was significantly predicted by Child Inhibition Score. Model fits are shown in the solid red line with dotted red lines indicating 95% confidence intervals. ^*^ *p* < 0.05, ^**^ *p* < 0.01.

## Data Availability

The data are not publicly available due to privacy or ethical restrictions but are available upon request from the corresponding author.
