# Supplementary material for: You Are Always on My Mind: Neural Synchrony Between Mothers and Their 2‐Year‐Olds During Collaborative Play
Source: Dev Sci. 2026 Jul 6;29(5):e70245. doi: 10.1111/desc.70245 (PMC13334935; doi:10.1111/desc.70245)
Supplement: Supplementary file 1 — Supporting Information: Table S1: Grouping of Channels for Region of Interest Analysis. Table S2: Long channel inclusion data for the mother and child. Table S3: Estimates of fixed effects for the HbO model M2: Coherence ∼ Condition + (1|Dyad). Table S4: Estimates of fixed effects for the HbO model M3: Coherence ∼ Condition + ROI + Condition x ROI + (1|Dyad). Table S5: Estimates of fixed effects for the HbR model M2: Coherence ∼ Condition + (1|Dyad). Table S6: Descriptive statistics for all additional participant variables. Table S7: Estimates of fixed effects for the HbO model M2.1: Coherence ∼ Condition + Child Age + (1|Dyad). Table S8: Estimates of fixed effects for the HbO model M2.2: Coherence ∼ Condition + MORS Invasiveness Score + (1|Dyad). Table S9: Estimates of fixed effects for the HbO model M2.3: Coherence ∼ Condition + Helping Score + (1|Dyad). Table S10: Estimates of fixed effects for the HbO model M4: Collaboration Coherence ∼ Inhibition Score + (1|Dyad). Figure S1: Factors that significantly predicted dyadic coherence across conditions and in Collaboration only. (a–c) Dyadic coherence across conditions was significantly predicted by (a) Child Age, (b) MORS Invasiveness Score and (c) Child Helping Score. (d) Dyadic coherence during the Collaboration condition was significantly predicted by Child Inhibition Score. Model fits are shown in the solid red line with dotted red lines indicating 95% confidence intervals. * p < 0.05, ** p < 0.01. [file DESC-29-e70245-s001.docx]

You Are Always on My Mind: Neural Synchrony Between Mothers and Their 2-Year-Olds During Collaborative Play - Supplementary Material

**METHOD**

**Materials**

Questionnaires

Mothers were asked to complete two questionnaires before the study – the *Mothers Object Relation Scale – Child version* (MORS- Child, Simkiss et al., 2013) and the *Ages and Stages 3rd Edition* (ASQ-3, Squires et al., 2009). The MORS-Child contains 2 subscales that provide a score of the mother’s perceived child warmth and child invasiveness by rating the commonness of certain child behaviours using a 6-point scale (from 0 – never to 5 – always). This questionnaire was included to assess the self-perceived mother-child relationship and to see how this perception may be associated with the dyad’s collaborative skills and neural synchrony.

The ASQ-3 is broken down into 3-month age brackets and contains sub-scales for communication, gross motor skills, fine motor skills, problem solving, and personal/social skills. Parents rate whether their child can perform the tasks/behaviours in the sub-scales (10 – yes, 5 – sometimes, not yet - 0). The ASQ-3 was included to ensure that all child participants were classed as typically developing. As most children were rated at ceiling for all subscales, these scores were ultimately not included in any further analysis.

**Additional Procedures**

Circle Drawing Inhibition Task

An adapted version of the Circle Drawing Task (Gandolfi et al., 2014) was used to provide a measure of inhibitory control and general executive function for the child. During this task, children were presented with an image of a circular track and two small toy cars. They were instructed to move the first car around the track at a normal speed and the second as slowly as possible. An ‘inhibition score’ was calculated for each child using the “inhibited time” / “normal time”. If the child showed no difference between the normal and inhibited tasks, they were given a score of 1.

Instrumental Helping Task

A helping task similar to the one described by Newton et al. (2016) was used to provide a measure of the child’s pro-social behaviour. Children were presented with a small toy dog. The experimenter would walk the dog across the table and then drop it out of their reach, next to the child. The experimenter would continue to look at the dropped toy, alternating with looks to the child after 10 seconds. If the child had not picked up the toy after 20 seconds, it was left, and the experimenter would move on. The child’s behaviour during the helping task was recorded and scored using the coding scheme by Newton et al. (2016): 0 indicated no interest in the dropped toy, and 5 indicated a successful completion where the child picked up the toy and returned it to the experimenter.

**Additional Statistical Analyses**

**Table S1. Grouping of Channels for Region of Interest Analysis**

|  | **Child ROI** | **Mother ROI** | **Child Channels Included** | **Mother Channels Included** |
| --- | --- | --- | --- | --- |
| **ROI 1** | Left PFC | Left PFC | 1,2,4,5 | 1,2,4,5 |
| **ROI 2** | Left PFC | Right PFC | 1,2,4,5 | 6,7,8,9 |
| **ROI 3** | Left PFC | Left TPJ | 1,2,4,5 | 10,11,12,13 |
| **ROI 4** | Left PFC | Right TPJ | 1,2,4,5 | 14,15,17,18 |
| **ROI 5** | Right PFC | Left PFC | 6,7,8,9 | 1,2,4,5 |
| **ROI 6** | Right PFC | Right PFC | 6,7,8,9 | 6,7,8,9 |
| **ROI 7** | Right PFC | Left TPJ | 6,7,8,9 | 10,11,12,13 |
| **ROI 8** | Right PFC | Right TPJ | 6,7,8,9 | 14,15,17,18 |
| **ROI 9** | Left TPJ | Left PFC | 10,11,12,13 | 1,2,4,5 |
| **ROI 10** | Left TPJ | Right PFC | 10,11,12,13 | 6,7,8,9 |
| **ROI 11** | Left TPJ | Left TPJ | 10,11,12,13 | 10,11,12,13 |
| **ROI 12** | Left TPJ | Right TPJ | 10,11,12,13 | 14,15,17,18 |
| **ROI 13** | Right TPJ | Left PFC | 14,15,17,18 | 1,2,4,5 |
| **ROI 14** | Right TPJ | Right PFC | 14,15,17,18 | 6,7,8,9 |
| **ROI 15** | Right TPJ | Left TPJ | 14,15,17,18 | 10,11,12,13 |
| **ROI 16** | Right TPJ | Right TPJ | 14,15,17,18 | 14,15,17,18 |

Note. PFC = Prefrontal cortex, TPJ = Temporoparietal Junction. Short separation channels 3 and 16 were not included in this nesting procedure.

Identification of Factors that Predict HbO Coherence in Mother-Toddler Dyads

Building on those reported in the Statistical Analyses in the main text, we constructed additional linear mixed-effect models to identify factors that may predict HbO coherence. Models were constructed using the coherence values from all regions of interest from both conditions combined, and with the coherence from the Collaboration condition only. The following additional variables were entered as fixed effects: child age, MORS Warmth score, MORS Invasiveness score, Child Inhibition score and Child Helping score.

Models were compared to either the experimental model: Coherence ~ Condition + (1|Dyad) or the null model: Collaborative Coherence ~ 1 + (1|Dyad) using ANOVAs with a Satterthwaite approximation for degrees of freedom.

**RESULTS**

**Table S2. Long channel inclusion data for the mother and child.**

|  | **Mean** | **SD** | **Min** | **Max** |
| --- | --- | --- | --- | --- |
| **Mother Channels** | 7.80 | 3.11 | 3 | 16 |
| **Child Channels** | 9.00 | 2.93 | 3 | 14 |

Note. Table S1 shows the long channel inclusion for the mother and child out of a possible 16 channels. Ten of the mothers and 5 of the children had one valid SSC. No participants had valid channels for both SSCs.

**S3. Estimates of fixed effects for the HbO model M2:**

**Coherence ~ Condition + (1|Dyad)**

| **Fixed Effects** | | | | | | | |
| --- | --- | --- | --- | --- | --- | --- | --- |
|  | *Estimate* | *SE* | *95% CI* | | *df* | *t* | *p* |
| Intercept | 0.324 | 0.001 | [0.322,0.326] | | 2918 | 271.1 |  |
| Condition | -3x10^-6^ | 0.002 | [-0.003, 0.003] | | 2918 | -0.002 | 0.999 |
| **Model Fit** | | | | | | | |
| *R^2^ (Adjusted)* | | | | *Maximum Likelihood Estimate* | | | |
| 0.0003 | | | | 4868.9 | | | |

**S4. Estimates of fixed effects for the HbO model M3:**

**Coherence ~ Condition + ROI + Condition x ROI + (1|Dyad)**

| **Fixed Effects** | | | | | | | |
| --- | --- | --- | --- | --- | --- | --- | --- |
|  | *Estimate* | *SE* | *95% CI* | | *df* | *t* | *p* |
| Intercept | 0.328 | 0.006 | [0.316, 0.34] | | 2888 | 55.4 |  |
| Collaboration | 0.005 | 0.008 | [-0.011, 0.022] | | 2888 | 0.61 | 0.541 |
| ROI 2 | -0.002 | 0.008 | [-0.017, 0.014] | | 2888 | -0.21 | 0.833 |
| ROI 3 | 0.003 | 0.008 | [-0.012, 0.018] | | 2888 | 0.33 | 0.739 |
| ROI 4 | -0.015 | 0.008 | [-0.031, 0.0005] | | 2888 | -1.90 | 0.057 |
| ROI 5 | -0.005 | 0.008 | [-0.021, 0.012] | | 2888 | -0.57 | 0.569 |
| ROI 6 | -0.009 | 0.008 | [-0.025, 0.006] | | 2888 | -1.16 | 0.245 |
| **ROI 7** | **0.016** | **0.008** | **[0.001, 0.031]** | | **2888** | **2.11** | **0.035*** |
| ROI 8 | -0.003 | 0.008 | [-0.019, 0.012] | | 2888 | -0.42 | 0.674 |
| ROI 9 | -0.003 | 0.008 | [-0.018, 0.013] | | 2888 | -0.33 | 0.741 |
| ROI 10 | -0.006 | 0.008 | [-0.021, 0.009] | | 2888 | -0.79 | 0.432 |
| ROI 11 | -0.011 | 0.007 | [-0.025, 0.003] | | 2888 | -1.59 | 0.113 |
| **ROI 12** | **-0.017** | **0.007** | **[-0.031, -0.003]** | | **2888** | **-2.33** | **0.020*** |
| ROI 13 | -0.011 | 0.008 | [-0.03, 0.004] | | 2888 | -1.46 | 0.144 |
| ROI 14 | -0.004 | 0.007 | [-0.018, 0.010] | | 2888 | -0.56 | 0.577 |
| ROI 15 | 0.004 | 0.007 | [-0.009, 0.018] | | 2888 | 0.65 | 0.519 |
| ROI 16 | -0.004 | 0.007 | [-0.018, 0.010] | | 2888 | -0.52 | 0.605 |
| Collaboration x ROI 2 | -0.003 | 0.011 | [-0.025, 0.020] | | 2888 | -0.23 | 0.817 |
| Collaboration x ROI 3 | -0.017 | 0.011 | [-0.038, 0.004] | | 2888 | -1.55 | 0.122 |
| Collaboration x ROI 4 | 0.016 | 0.011 | [-0.006, 0.039] | | 2888 | 1.42 | 0.156 |
| Collaboration x ROI 5 | -0.003 | 0.012 | [-0.026, 0.020] | | 2888 | -0.22 | 0.823 |
| Collaboration x ROI 6 | -0.009 | 0.011 | [-0.032, 0.013] | | 2888 | -0.79 | 0.427 |
| **Collaboration x ROI 7** | **-0.037** | **0.011** | **[-0.058, -0.016]** | | **2888** | **-3.46** | **0.001**** |
| Collaboration x ROI 8 | -0.011 | 0.011 | [-0.033, 0.012] | | 2888 | -0.94 | 0.345 |
| Collaboration x ROI 9 | -0.006 | 0.011 | [-0.028, 0.015] | | 2888 | -0.57 | 0.566 |
| Collaboration x ROI 10 | 0.002 | 0.011 | [-0.019, 0.023] | | 2888 | 0.20 | 0.845 |
| Collaboration x ROI 11 | 0.006 | 0.010 | [-0.014, 0.026] | | 2888 | 0.62 | 0.537 |
| Collaboration x ROI 12 | 0.004 | 0.010 | [-0.016, 0.024] | | 2888 | 0.40 | 0.689 |
| Collaboration x ROI 13 | -0.002 | 0.011 | [-0.023, 0.019] | | 2888 | -0.18 | 0.855 |
| Collaboration x ROI 14 | 0.001 | 0.010 | [-0.019, 0.021] | | 2888 | 0.10 | 0.919 |
| Collaboration x ROI 15 | -0.018 | 0.010 | [-0.038, 0.0009] | | 2888 | -1.87 | 0.062 |
| Collaboration x ROI 16 | -0.001 | 0.010 | [-0.021, 0.019] | | 2888 | -0.14 | 0.891 |
| **Model Fit** | | | | | | | |
| *R^2^ (Adjusted)* | | | | *Maximum Likelihood Estimate* | | | |
| 0.0138 | | | | 4904.8 | | | |

Note. Significant fixed effects are shown in bold. * p<.05, **p<.01.

**S5. Estimates of fixed effects for the HbR model M1:**

**Coherence ~ Condition + (1|Dyad)**

| **Fixed Effects** | | | | | | | |
| --- | --- | --- | --- | --- | --- | --- | --- |
|  | *Estimate* | *SE* | *95% CI* | | *df* | *t* | *P* |
| Intercept | 0.321 | 0.001 | [0.318, 0.323] | | 2918 | 262.6 |  |
| **Condition** | **-0.004** | **0.002** | **[-0.007, -0.0006]** | | **2918** | **-2.32** | **0.021*** |
| **Model Fit** | | | | | | | |
| *R^2^ (Adjusted)* | | | | *Maximum Likelihood Estimate* | | | |
| 0.0015 | | | | 4807.5 | | | |

Note. Significant fixed effects are shown in bold. * p<.05, **p<.01.

**Identification of Factors that Predict HbO Coherence in Mother-Toddler Dyads:**

**Table S6. Descriptive statistics for all additional participant variables.**

|  | **Mean** | **SD** | **N** |
| --- | --- | --- | --- |
| Child Age (months) | 30.15 | 2.85 | 20 |
| MORS-Child Warmth | 28.85 | 2.81 | 20 |
| MORS-Child Invasiveness | 12.05 | 5.18 | 20 |
| Inhibition Score | 2.11 | 1.69 | 14 |
| Helping Score | 2.31 | 1.66 | 16 |

Note. Six children did not complete the inhibition task, and 4 did not complete the helping task during their sessions. MORS = Mother Object Relation Scale questionnaire.

Factors Predicting Coherence Across Conditions

The addition of the following fixed effects significantly improved the model over HbO M1, after correction for multiple comparisons: Child Age (*χ^2^*(1) = 13.8, *p* <.001), MORS Invasiveness Score (*χ^2^*(1) = 5.91, *p* =.015) and Helping Score (*χ^2^*(1) = 8.94, *p*=.003). Full estimates of fixed effects can be found in the supplementary tables S7-S10.

HbO coherence across conditions was negatively predicted by increased child age and higher MORS Invasiveness scores (shown in Figures S1a-b). However, HbO coherence was positively predicted by higher child Helping scores (Figure S1c).

Factors Predicting Collaboration Coherence

Only the child Inhibition Score significantly predicted coherence for the Collaboration condition, with higher Inhibition Scores negatively predicting coherence, as shown in Figure S1d.

Figure S1. Factors that significantly predicted dyadic coherence across conditions and in Collaboration only. (a-c) Dyadic coherence across conditions was significantly predicted by (a) Child Age, (b) MORS Invasiveness Score and (c) Child Helping Score. (d) Dyadic coherence during the Collaboration condition was significantly predicted by Child Inhibition Score. Model fits are shown in the solid red line with dotted red lines indicating 95% confidence intervals. * p<.05, ** p<.01**
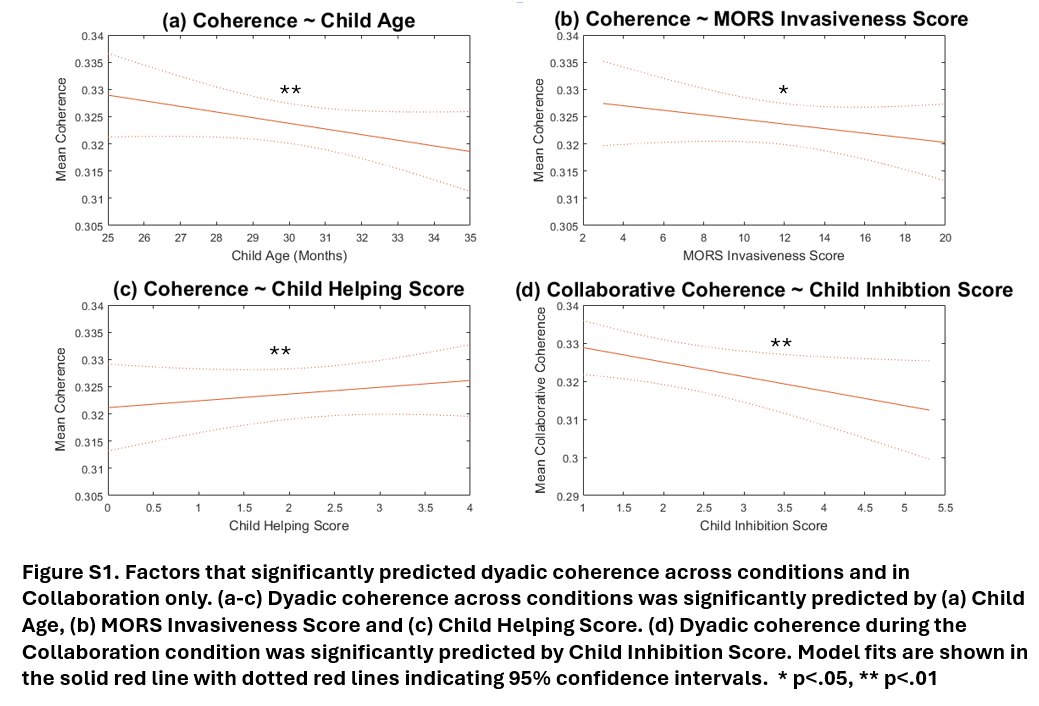
**

**S7. Estimates of fixed effects for the HbO model M3.1:**

**Coherence ~ Condition + Child Age + (1|Dyad)**

| **Fixed Effects** | | | | | | | |
| --- | --- | --- | --- | --- | --- | --- | --- |
|  | *Estimate* | *SE* | *95% CI* | | *df* | *t* | *P* |
| Intercept | 0.358 | 0.009 | [0.340, 0.376] | | 2917 | 38.7 |  |
| Condition | -3x10^-6^ | 0.002 | [-0.003, 0.003] | | 2917 | -0.002 | 0.999 |
| **Child Age** | **-0.001** | **0.0003** | **[-0.002, -0.0005]** | | **2917** | **-3.73** | **<.001**** |
| **Model Fit** | | | | | | | |
| *R^2^ (Adjusted)* | | | | *Maximum Likelihood Estimate* | | | |
| 0.0041 | | | | 4875.8 | | | |

Note. Significant fixed effects are shown in bold. * p<.05, **p<.01.

**S8. Estimates of fixed effects for the HbO model M3.2:**

**Coherence ~ Condition + MORS Invasiveness Score + (1|Dyad)**

| **Fixed Effects** | | | | | | | |
| --- | --- | --- | --- | --- | --- | --- | --- |
|  | *Estimate* | *SE* | *95% CI* | | *df* | *t* | *P* |
| Intercept | 0.329 | 0.0023 | [0.324, 0.333] | | 2917 | 144.3 |  |
| Condition | -3x10^-6^ | 0.0017 | [-0.003, 0.003] | | 2917 | -0.002 | 0.999 |
| **MORS I** | **-0.0004** | **0.00016** | **[-0.0007, -8x10^-5^]** | | **2917** | **-2.43** | **0.015*** |
| **Model Fit** | | | | | | | |
| *R^2^ (Adjusted)* | | | | *Maximum Likelihood Estimate* | | | |
| 0.0013 | | | | 4871.8 | | | |

Note. Significant fixed effects are shown in bold. * p<.05, **p<.01.

**S9. Estimates of fixed effects for the HbO model M3.3:**

**Coherence ~ Condition + Helping Score + (1|Dyad)**

| **Fixed Effects** | | | | | | | |
| --- | --- | --- | --- | --- | --- | --- | --- |
|  | *Estimate* | *SE* | *95% CI* | | *df* | *t* | *P* |
| Intercept | 0.320 | 0.002 | [0.317, 0.324] | | 2631 | 190.2 | 0 |
| Condition | 0.0009 | 0.002 | [-0.003, 0.004] | | 2631 | 0.51 | 0.61 |
| **Helping Score** | **0.0017** | **0.0006** | **[0.006, 0.003]** | | **2631** | **2.99** | **0.003**** |
| **Model Fit** | | | | | | | |
| *R^2^ (Adjusted)* | | | | *Maximum Likelihood Estimate* | | | |
| 0.0027 | | | | 4406.2 | | | |

Note. Significant fixed effects are shown in bold. * p<.05, **p<.01.

**S10. Estimates of fixed effects for the HbO model M4:**

**Collaboration Coherence ~ Inhibition Score + (1|Dyad)**

| **Fixed Effects** | | | | | | | |
| --- | --- | --- | --- | --- | --- | --- | --- |
|  | *Estimate* | *SE* | *95% CI* | | *df* | *t* | *P* |
| Intercept | 0.337 | 0.002 | [0.333, 0.341] | | 1022 | 157.3 |  |
| **Inhibition Score** | **-0.005** | **0.0008** | **[-0.007, -0.004]** | | **1022** | **-6.45** | **<.001**** |
| **Model Fit** | | | | | | | |
| *R^2^ (Adjusted)* | | | | *Maximum Likelihood Estimate* | | | |
| 0.0381 | | | | 1772.8 | | | |

Note. Significant fixed effects are shown in bold. * p<.05, **p<.01.

**REFERENCES**

Gandolfi, E., Viterbori, P., Traverso, L., & Usai, M. C. (2014). Inhibitory processes in toddlers: A latent-variable approach. *Frontiers in Psychology*, *5*. https://doi.org/10.3389/fpsyg.2014.00381

Newton, E. K., Thompson, R. A., & Goodman, M. (2016). Individual Differences in Toddlers’ Prosociality: Experiences in Early Relationships Explain Variability in Prosocial Behavior. *Child Development*, *87*(6), 1715–1726. https://doi.org/10.1111/cdev.12631

Simkiss, D. E., MacCallum, F., Fan, E. E. Y., Oates, J. M., Kimani, P. K., & Stewart-Brown, S. (2013). Validation of the mothers object relations scales in 2–4 year old children and comparison with the child–parent relationship scale. *Health and Quality of Life Outcomes*, *11*(1). https://oro.open.ac.uk/37587/

Squires, J., Bricker, D., & Twombly, E. (2009). *Ages & Stages Questionnaires. 3rd Ed.* Brooks Publishing Company.
